# A Programmable Liquid Crystal Elastomer Metamaterials With Soft Elasticity

**DOI:** 10.3389/frobt.2022.849516

**Published:** 2022-02-25

**Authors:** Xudong Liang, Dongfeng Li

**Affiliations:** School of Science, Harbin Institute of Technology (Shenzhen), Shenzhen, China

**Keywords:** metamaterials, liquid crystal elastomer, soft elasticity, programmable materials, strain softening and stiffening

## Abstract

Liquid crystal elastomers (LCEs) are a rubbery network of polymers with ordered liquid crystal mesogens. The combination of rubber elasticity and the anisotropic liquid crystalline order gives exceptional mechanical properties, like soft elasticity, where near-constant stress accompanies large elastic deformation in the material. However, the soft elasticity in LCEs is often bounded by the intrinsic molecular interactions and structures, limiting the range of programmable mechanical properties and functionalities. Here, we demonstrate that the semi-soft elasticity of LCEs can be integrated into the framework of metamaterials to realize markedly programmabilities. Under uniaxial deformation, each state of the building blocks in metamaterials and the molecular composition of the nematic LCEs is associated with a distinctly different stress-strain relation that is fully elastic. Taking advantage of the tunable bending and stretching deformation enabled by the geometry of the building blocks and the semi-soft elasticity of the nematic LCE in the metamaterials, we can engineer the local stretch and stress at an unmet level of their counterpart composed by elastomers. Numerical simulations and analytical models are developed to relate the metamaterial geometries and the LCE soft elasticity to the mechanical responses. In addition, an elastic region with near-zero stiffness up to a stretch of 1.4 can be designed by connecting the compliant responses due to bending deformation and the soft elasticity in the LCE. We expect that the specialized mechanical tunability enabled by the LCE metamaterials can facilitate the development of advanced forms of mechanical metamaterials and impact the design of robotic systems.

## 1 Introduction

Programming mechanical properties and functionalities are one of the most fundamental goals in material science, owing to its central role in applications (Surjadi et al., 2019). To design a material with a targeted set of properties and mechanical responses, people have endeavored to discover new materials, modify the material compositions, and optimize the material manufacturing process (Meyers and Chawla, 2008; Fleck et al., 2010). Great progress has been made in developing materials with the desired properties over the past centuries. However, intrinsic mechanical properties and the basic physical mechanisms often limit the capacity to design materials with desired responses (Surjadi et al., 2019). For example, the positive stiffness is required for stability of an unconstrained block of material in deformation based on thermodynamics (Wang and Lakes, 2005; Coulais et al., 2015); the stiffness-toughness conflict exists in regular polymer networks where the crosslinks stiffen the polymers but embrittle them (Lake and Thomas, 1967; Kim et al., 2021); the intimate coupling between strength and density is observed in most materials where high strength normally means large density (Schaedler et al., 2011; Yu et al., 2018).

Nature has provided numerous examples of well-defined architectures to bypass the general limitations in material design, achieving mechanical properties and functionalities differing from and surpassing constituent materials (Meyers et al., 2008; Launey et al., 2010). For example, the carps are known to have superior toughness against the penetration from predator’s tooth by developing the “Bouligand structure” in their scales, where fibrils of collagen are aligned in layers, with the fibril in each layer rotating by roughly 36° (Quan et al., 2020). Soft biological tissues composed of semiflexible filamentous proteins can undergo strain-stiffening, with a tenfold increase in shear moduli under strains as small as 20% (Gardel et al., 2004; Storm et al., 2005).

Inspired by the architectures in biological materials, recent advancements in manufacturing techniques have enabled material design via geometric arrangements of the underlying structures (Bertoldi et al., 2017; Kadic et al., 2019). Built upon the periodically arranged building blocks, mechanical metamaterial has been fabricated to mediate mechanical deformation, stress, and energy (Kochmann and Bertoldi, 2017). It also boasts the discoveries of functionalities not available in natural materials, such as auxeticity (Lakes, 1987), negative stiffness (Coulais et al., 2015), and non-monotonic energy dissipations (Liang and Crosby, 2020a). Traditionally, the design for properties exhibited in metamaterials relies on the structural responses in the periodic building blocks, where a linear elastic or a hyperelastic constitutive relation of the materials are adopted (Bertoldi et al., 2017; Pishvar and Harne, 2020). Recently, new characteristics have been realized in metamaterials by combining additional fields beyond elasticity. For example, the ability to alter mechanical memories with stable memories (Chen et al., 2021), or to generate reversible solid-solid phase transitions (Liang et al., 2022) is achieved by incorporating magnetic domains in metamaterials. However, these functionalities are still programmed through the building blocks’ geometries, focusing on the spatial heterogeneity in the structures.

The control of the structural responses of the building blocks has motivated the design of sophisticated layouts in the metamaterials while avoiding selecting materials with complex constitutive responses in previous studies. Although effective, such a paradigm might constrain the tunability of the mechanical properties and functionalities, leaving an ample design space unexplored for the material-based programmability. Synergetically combining the material-level constitutive behaviors and structural-level building block mechanical responses can open new avenues for programmable metamaterials. A recent study has demonstrated that coupling the constitutive materials’ viscoelasticity with the elastic snap-through instability in metamaterials can exhibit a programmable hysteric response with optimal dissipations at different loading rates (Dykstra et al., 2019).

In this paper, we integrate the soft elasticity in the liquid crystal elastomer (LCE) into the framework of metamaterials. By engraving the orthogonally aligned elliptical pores into an LCE sheet, we can create a metamaterial with networks of “plates” connected by thin “ligaments” that undergo programmable strain-softening to strain-stiffening responses (Liang and Crosby, 2020b). The nematic LCE in the metamaterials can enter the state of soft elasticity as the material is stretched vertically to the liquid crystal ordering direction, where the deformation is accommodated with near-constant stress as liquid crystal mesogens rotate (Küupfer and Finkelmann, 1994; Warner and Terentjev, 2007; Biggins et al., 2008). As a result, the nematic LCEs have a “stress-strain plateau” during liquid crystal mesogens’ rotation and stiffen as the molecules align to the stretching direction. Although the constitutive responses in LCEs rely on the molecular structures, the critical stretch to trigger the compliant and stiffening effect is controlled by the local deformation in the building blocks (Liang and Crosby, 2020b). By tuning the structural geometry and the molecular compositions of the metamaterials, we can design the structural responses in the building blocks and the soft elasticity of the LCEs in a coupled and controllable manner. This framework can extend the design space in the material-based tunability unavailable by controlling the geometry in the building blocks alone.

This paper studies the mechanical responses of metamaterials composed of liquid crystal elastomers with soft elasticity. The paper is structured as follows. We first describe the semi-soft elasticity constitutive model of the nematic LCEs stretched perpendicularly to the molecule’s ordering direction in Section 2. Finite element simulations that incorporate the semi-soft elasticity constitutive model into metamaterials with different geometries in the building blocks are presented in Section 3. The simulated mechanical responses reflect how the soft elasticity in LCEs and the pore shapes in the building block synergetically program the mechanical responses, particularly the strain-softening and strain-stiffening behaviors. In Section 4, we derive a reduced analytical model to understand the coupling between the material and the structural properties. The metamaterial is modeled as rigid rotating plates connected by elastic springs, in which the soft elasticity governs the material constitutive responses. We conclude with a few remarks on material-based tunability based on the metamaterial geometry and the soft elasticity, with a new strategy for programming material properties and functionalities.

## 2 Neo-Classical Theory for LCEs

Liquid crystal elastomers are rubbery polymer networks composed of molecules with liquid-like mobility and are capable of withstanding large deformation. By incorporating the spontaneous liquid-crystalline ordering into networks of the polymers, the nematic LCEs can reach a delicate balance between the stiffness of the liquid crystal molecules and the entropically driven elasticity of polymer chains (Warner and Terentjev, 2007). Compared to elastomers described by the classical rubber elasticity, the aligned liquid crystal mesogens in the nematic LCE induce the molecular shape anisotropy, modifying the elastic function in deformation (Bladon et al., 1993; Verwey and Warner, 1997a). Previous efforts have been made to extend the classical rubber elasticity to account for the nuances with liquid crystal orderings, known as the “neo-classical” theory for rubber elasticity (Warner and Terentjev, 2007; Biggins et al., 2008; Biggins et al., 2012; Sonnet and Virga, 2012). Here, we briefly introduce the “neo-classical” theory of nematic LCEs for the completeness of the current study. More systematic considerations can be found in Warner and Terentjev (2007). In addition, the viscoelasticity of the polymer network and the rate dependence of the mesogen director rotation can substantially affect the constitutive responses of the nematic LCE (Warner and Terentjev, 2007; Zhang et al., 2019). Here, we focus on the quasi-static responses of the LCE metamaterial and model the nematic LCE with the “neo-classical” theory.

**FIGURE 1:**
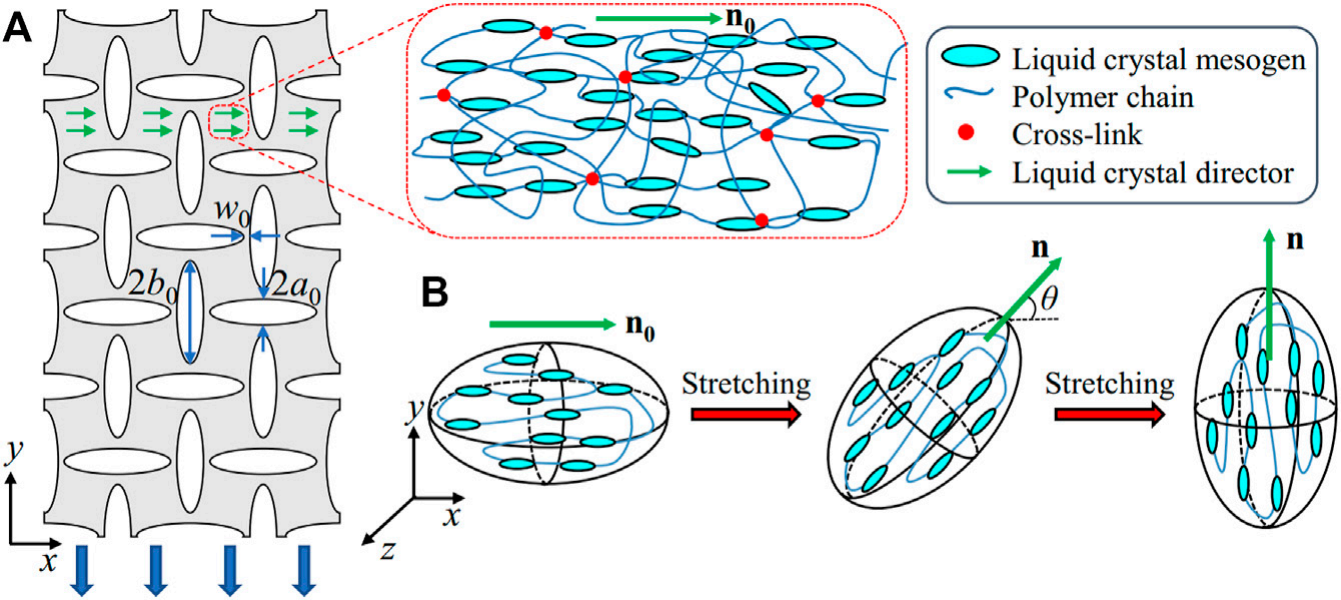
Schematics of the LCE metamaterials. **(A)** The metamaterials composed of the nematic LCE aligned along the *x*-axis, with engraved pores. The metamaterial is stretched along the *y*-axis, orthogonal to the liquid crystal ordering. The nematic LCE is a network of crosslinked polymer chains with aligned liquid crystal mesogens (indicated by the director **n**_**0**_). **(B)** The ellipsoid formed between the crosslinks in the LCE polymer chains due to the liquid crystalline ordering. With the vertical extension, the ellipsoid and the liquid crystal director rotate with an angle *θ* along the *z*-axis. The rotation is completed as the liquid crystal mesogens are aligned along the *y*-axis, with *θ*=π/2.

The liquid crystal mesogens are aligned along the *x*-axis in fabrications, as indicated by the director **n**_**0**_ with the green arrows in Figure 1A. The presence of liquid crystal mesogens in the polymer networks leads to an anisotropic Gaussian distribution of the end-to-end vector of the polymer chain between the two crosslinks, **R**. The mean square end-to-end vector for the polymer chain follows,

$$\begin{array}{c} <R_{i}R_{j}>=\frac{1}{3}l_{ij}L,\;\;i,j=1,\cdots ,3 \end{array}$$ (1)

where ***l*** = *l*_*ij*_ is the effective step length tensor for the nematic LCE; *L* is the arclength of the polymer chain, where *L* = *Nb*, with *N* being the number of monomers between two crosslinks and *b* being the step length of the monomer. The bracket <..> represents the averaging for *N* monomers in the polymer chain. In the undeformed nematic LCEs, the director **n**_**0**_ is along the *x*-axis, and the mean square sizes in the plane perpendicular to the **n**_**0**_ are identical. Therefore, the step length tensor at the undeformed state can be written as,

$$\begin{array}{c} l_{0}=\left(\begin{array}{ccc} l_{n} & 0 & 0\\ 0 & l_{p} & 0\\ 0 & 0 & l_{p} \end{array}\right), \end{array}$$ (2)

where *l*_*n*_ and *l*_*p*_ are the effective step lengths in the directions parallel and perpendicular, respectively, to the liquid crystal director **n**_**0**_. The anisotropic step lengths in Eq. 2 turn the spheroid formed by the polymer chain in elastomers into an ellipsoid in the LCEs (Herbert et al., 2021), as shown in Figure 1B. The ratio between the two effective step lengths, *r* = *l*_*n*_/*l*_*p*_, representing the anisotropy for the nematic LCE, can be further expressed with the nematic order of the liquid crystal ordering, *Q*,

$$\begin{array}{c} r=\frac{1+2Q}{1-Q}, \end{array}$$ (3)

where *Q* = 1 represents the perfect nematic ordering with the liquid crystal mesogens directed in one direction, and *Q* = 0 corresponds to randomly oriented mesogens (Warner and Terentjev, 2007; Sonnet and Virga, 2012).

As the nematic LCEs aligned along the horizontal direction are stretched along the *y*-axis, the liquid crystal mesogens anchoring to the bulk rotate along the *z*-axis with an angle *θ* (Figure 1B). The step length tensor in the deformed state is now written as,

$$\begin{array}{c} l=\left(\begin{array}{ccc} l_{n}+\left(l_{p}-l_{n}\right){\text{sin}}^{2}\theta & \left(l_{p}-l_{n}\right)\text{sinθcosθ} & 0\\ \left(l_{p}-l_{n}\right)\text{sinθcosθ} & l_{p}-\left(l_{p}-l_{n}\right){\text{sin}}^{2}\theta & 0\\ 0 & 0 & l_{p} \end{array}\right). \end{array}$$ (4)

Considering the entropy changes of crosslinked polymer chains with liquid crystal mesogens in deformation, the free energy density of the nematic LCEs is (Bladon et al., 1993; Warner and Terentjev, 2007),

$$W_{el}=\frac{1}{2}\mu Tr\left(l_{0}\cdot F^{\text{T}}\cdot l^{-1}\cdot F\right),$$ (5)

where **F** is the deformation gradient for the LCE and *µ* is the small-strain shear modulus of the nematic LCE. To account for variations in the anisotropy *r* due to compositional fluctuations in the liquid crystal ordering, an additional energy term that leads to “semi-softness” in rotation (Verwey and Warner, 1997b; Popov and Semenov, 1998) is introduced,

$$W_{ss}=\frac{1}{2}\alpha \mu Tr\left(\left(I-n_{0}n_{0}\right)\cdot F^{T}\cdot nn\cdot F\right),$$ (6)

where α=<1/*r* > −1/<*r*> is the anisotropy fluctuation of the anisotropy of the nematic LCE, α > 0; **n** = cos*θ* **e**_**x**_ + sin*θ* **e**_**y**_ is the liquid crystal director at the deformed state, and **I** is the identity tensor. Therefore, the free energy density for LCEs is *W*=*W*_*el*_ + *W*_*ss*_ (Verwey and Warner, 1997a; Warner and Terentjev, 2007).

Considering the metamaterials with an extension imposed along the *y*-axis (Figure 1A), we follow the model of stretching strips of nematic LCEs perpendicular to the director (Verwey et al., 1996; Verwey and Warner, 1997a; Warner and Terentjev, 2007) by integrating the step length tensor in Eq. 4 into the free energy density functions in Eqs 5, 6. The director of the liquid crystal **n** rotates to the optimal value that minimizes the free energy density *W* in stretching, and the nematic LCE is relaxed on the plane normal to the *x*-axis, with the nominal stress components *S*_*xy*_ *= S*_*xx*_ *=* 0. Therefore, the free energy density of the LCEs is a function of the stretch in the *y*-axis, $\bar{\lambda }\;$ , and the director rotation angle, *θ*,

$$\begin{array}{c} W\left(\bar{\lambda },\theta \right)=\frac{1}{2}\mu \left[{\bar{\lambda }}^{2}\left(1-\frac{r-1}{r}{\text{sin}}^{2}\theta \right)+\frac{2}{\bar{\lambda }\sqrt{1-\frac{r-1}{r}{\text{sin}}^{2}\theta }}+\alpha {\bar{\lambda }}^{2}{\text{sin}}^{2}\theta \right]. \end{array}$$ (7)

Here we distinguish the local stretch in the material with a bar as $\bar{\lambda }$ . Therefore, the critical conditions for the liquid crystal mesogens’ rotation are obtained by ∂*W*/∂sin^2^ *θ* = 0, namely,

$$\begin{array}{c} \frac{r-1}{r}{\text{sin}}^{2}\theta =1-\frac{1}{{\bar{\lambda }}^{2}}{\left(\frac{r-1}{r-1-\alpha r}\right)}^{2/3}. \end{array}$$ (8)

From Eq. 8, we can obtain the critical stretch for the onset of rotation, ${\bar{\lambda }}_{1}$ =((*r*−1)/(*r*−1−α*r*))^1/3^, by setting *θ* = 0, and the critical stretch for the completion of rotation (Figure 1B, right), ${\bar{\lambda }}_{2}=\sqrt{r}{\bar{\lambda }}_{1}$ , by setting *θ* = π/2. Before the rotation of the liquid crystal mesogens, $\bar{\lambda }<{\bar{\lambda }}_{1}$ (Figure 1B, left), the nematic LCEs behave like the traditional elastomer, with *θ* = 0. As the stretch is between the threshold and the end for soft elasticity, ${\bar{\lambda }}_{1}<\bar{\lambda }<\sqrt{r}{\bar{\lambda }}_{1}$ (Figure 1B, middle), the liquid crystal mesogens rotate from *θ* = 0 to *θ* = π/2, where the rotational angle *θ* increases with the extension. For the stretch beyond $\sqrt{r}{\bar{\lambda }}_{1}$ (Figure 1B, right), the rotation of the liquid crystal mesogens is complete, with the director **n** aligning along the *y*-axis, and the nematic LCEs behave like the traditional elastomer again.

While sophisticated continuum constitutive models (Zhang et al., 2019) and computational micromechanics simulations (Zhou and Bhattacharya, 2021) for nematic LCEs have been proposed recently, we shall proceed with the simple phenomenological description of LCE with free energy density function in Eq. 7. The LCE metamaterials under the imposed uniaxial stretching deform in the thin ligament region, where the nematic LCEs are stretched along the *y*-axis and relaxed in the transverse dimension along the *x*-axis. Therefore, we only focus on the stress component along the *y*-axis, and the corresponding nominal stress follows, $S_{\mathrm{yy}}=\partial \mathrm{W/}\partial \bar{\lambda }$ . A microstructure of stripes with the oppositely rotated nematic director may develop during soft deformation in the ligament when the constrained boundaries prohibit the shear strain along the y-axis (Warner and Terentjev, 2007; Bai and Bhattacharya, 2020). However, in our study, the thin ligament is directly connected to the plate regions without constraining the shear strain. Therefore, the stripe domain microstructure will not emerge in the LCE metamaterial. The constitutive relation with respect to the three stages of soft elasticity is expressed by

$$\begin{array}{c} S_{yy}=\{\begin{array}{cc} \mu \left(\bar{\lambda }-\frac{1}{{\bar{\lambda }}^{2}}\right),\;\;\;\;\;\;\;\;\;\;\;\;\;\;\;\;\;\;\;\; & \bar{\lambda }<{\bar{\lambda }}_{1}\\ \mu \bar{\lambda }\left(1-\frac{1}{{\bar{\lambda }}_{1}^{3}}\right),\;\;\;\;\;\;\;\;\;\;\;\;\;\;\;\;\; & {\bar{\lambda }}_{1}<\bar{\lambda }<\sqrt{r}{\bar{\lambda }}_{1}\\ \mu \left(\bar{\lambda }\left(1-\frac{r-1}{{\bar{\lambda }}_{1}^{3}r}\right)-\frac{\sqrt{r}}{{\bar{\lambda }}^{2}}\right), & \bar{\lambda }>\sqrt{r}{\bar{\lambda }}_{1} \end{array}. \end{array}$$ (9)

**FIGURE 2:**
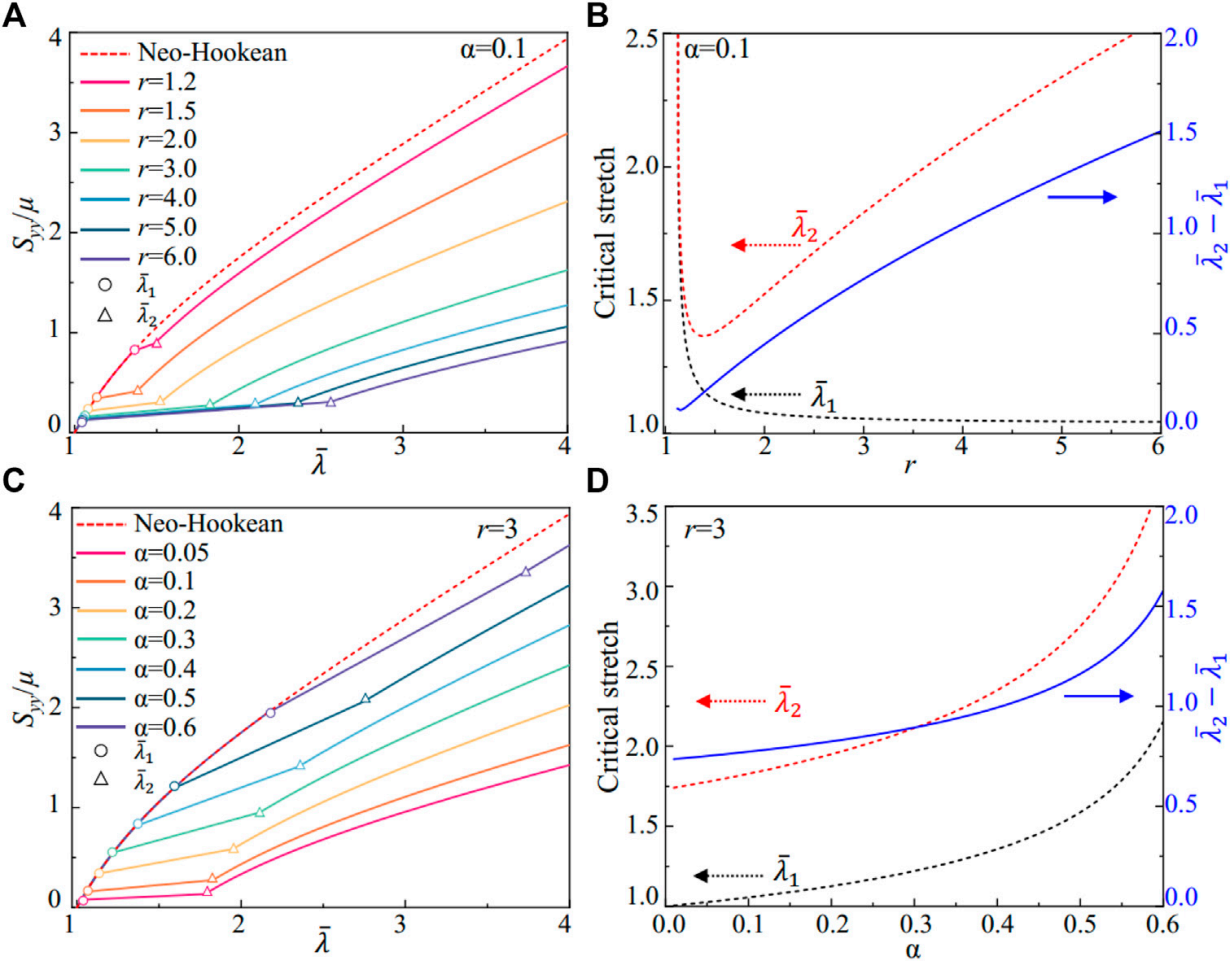
Constitutive responses of semi-soft elasticity of the nematic LCE. **(A)** The scaled nominal stress *S*_*yy*_/*μ*, and **(B)** the critical stretches for the soft elasticity in uniaxial extension with different liquid crystalline ordering *r* and a fixed fluctuation α=0.1. **(C)** The scaled nominal stress *S*_*yy*_/*μ*, and **(D)** the critical stretches for the soft elasticity in uniaxial extension with different liquid crystalline ordering fluctuations α and a fixed anisotropy *r*=3.

In Figure 2, we compare the scaled nominal stress of the nematic LCEs with soft elasticity predicted by Eq. 9 with the traditional elastomers described by the Neo-Hookean model. By controlling the ratio for the anisotropy *r* and the fluctuations of the anisotropy α, we can program the stress-strain relations in nematic LCEs under the uniaxial extension perpendicular to the liquid crystal alignment direction **n**_**0**_. In Figure 2A, we plot scaled nominal stress with the fluctuation fixed at α = 0.1 and the anisotropy of the nematic LCEs increasing from *r* = 1.5 to *r* = 6. The mechanical responses in the nematic LCE are the same Neo-Hookean elastomers for *r* = 1, and the liquid crystal alignment along with the *x*-axis increases as *r* increases from 1. The neo-classical theory captures the semi-soft elastic responses in LCEs as the stress becomes near constant with increasing stretch. The materials behave like the traditional elastomers as the stretch is below ${\bar{\lambda }}_{1}$ or above ${\bar{\lambda }}_{2}$ , reaching a semi-soft response as the liquid crystal mesogens rotate with ${\bar{\lambda }}_{1}$ < ${\bar{\lambda }}_{l}$ < ${\bar{\lambda }}_{2}$ . The nematic LCE shows a softer response than Neo-Hookean materials due to the rotation of liquid crystal mesogens. The anisotropy of the nematic LCEs (*r*), which is controlled by the liquid crystal ordering *Q* (Eq. 3), can further program the stress plateau with the critical stretches ${\bar{\lambda }}_{1}$ and ${\bar{\lambda }}_{2}$ . As shown in Figure 2B, the critical stretches grow infinitely at *r* = 1, where the Neo-Hookean model describes the stress in the nematic LCE, as indicated in Eq. 9. The critical stretch for the onset of the soft response $\left({\bar{\lambda }}_{1}\right)$ decreases with *r* rapidly, while the critical stretch for existing the soft response $\left({\bar{\lambda }}_{2}\right)$ grows with *r*, for *r* > 1.5. Therefore, the stretch for the semi-soft responses ${\bar{\lambda }}_{2}$ − ${\bar{\lambda }}_{1}$ increases with the liquid crystal ordering *r* (solid blue line in Figure 2B).

We can further design the softness responses via the fluctuation of the liquid crystal ordering in the nematic LCE. With a fixed anisotropy *r* = 3, the nematic LCE shows softness responses for different values of *α* (Figure 2C). As the fluctuation parameter *α* increases from 0, where there are no variations in the liquid crystal ordering, the stress in the LCE under the same local stretch $\bar{\lambda }$ increases with *α*. The nematic LCE’s mechanical responses are close to the Neo-Hookean materials as *α* = 0.6, where the fluctuation in the liquid crystal ordering requires additional energy for rotation, leading to the mechanical responses governed by the polymer chains, similar to the Neo-Hookean materials. This phenomenon is further verified in Figure 2D as the critical stretches approach infinity with increasing *α*, where the Neo-Hookean model describes the stress-strain relations up to a large local stretch ${\bar{\lambda }}_{1}$ in the nematic LCE (black dashed line in Figure 2D). In addition, a “stress-strain plateau” with finite stretch is observed in Figure 2C due to the fixed anisotropy of the nematic LCE with the same liquid crystal ordering. The semi-softness stretch ${\bar{\lambda }}_{2}$ − ${\bar{\lambda }}_{1}$ also increases with the fluctuations of the liquid crystal ordering *α*, possibly caused by the higher energy barrier and the larger resultant stress applied to the polymer network in deformation.

## 3 Numerical Models for LCE Metamaterials

**FIGURE 3:**
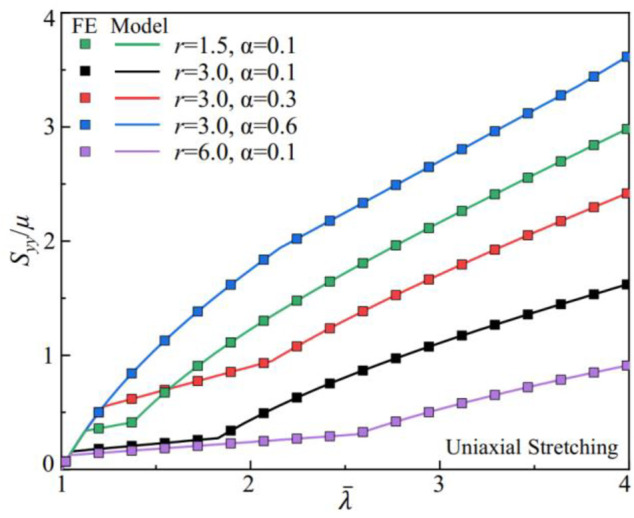
Stress-stretch relations of uniaxial stretching nematic LCEs. The solid lines are the predictions from the phenomenological model of soft elasticity in Eq. 9. The solid symbols are simulation results of the homogeneously stretched rectangular bars with the material model defined by the Marlow strain energy potential.

We conduct numerical simulations using finite element (FE) methods to fully explore the relationship between the metamaterial building blocks’ geometry and the nematic LCE’s constitutive responses, especially the connection between global deformation and the local soft elasticity. Quasi-static nonlinear analysis of the LCE metamaterials under uniaxial stretching is performed using the commercial FE software Abaqus. The constitutive responses of the nematic LCE are modeled with the Marlow model, where the uniaxial stress-stretch relation from Eq. 9 is used to define the strain energy potential in the simulations. In Figure 3, we compare the stress-strain relation defined by the Marlow model in the simulations of uniaxially stretched rectangular bars (solid symbols) to the one from Eq. 9 (solid lines). The deformation in the uniaxial stretching is homogeneous, ensuring that the local stretch in the nematic LCE is the same as the applied global stretch, $\bar{\lambda }=\lambda$ . The Marlow strain energy potential reproduces the constitutive responses of the nematic LCE with soft elasticity. The dependence of the stress-stretch relation upon the liquid crystal ordering *r* and its fluctuation *α* are correctly captured in the simulations with the Marlow model. Therefore, we model the inhomogeneous deformation in the LCE metamaterial with the Marlow model defined by the uniaxial stress-stretch relation from Eq. 9 and study the coupling between the soft elasticity in the nematic LCE and the geometry of the metamaterials.

**FIGURE 4:**
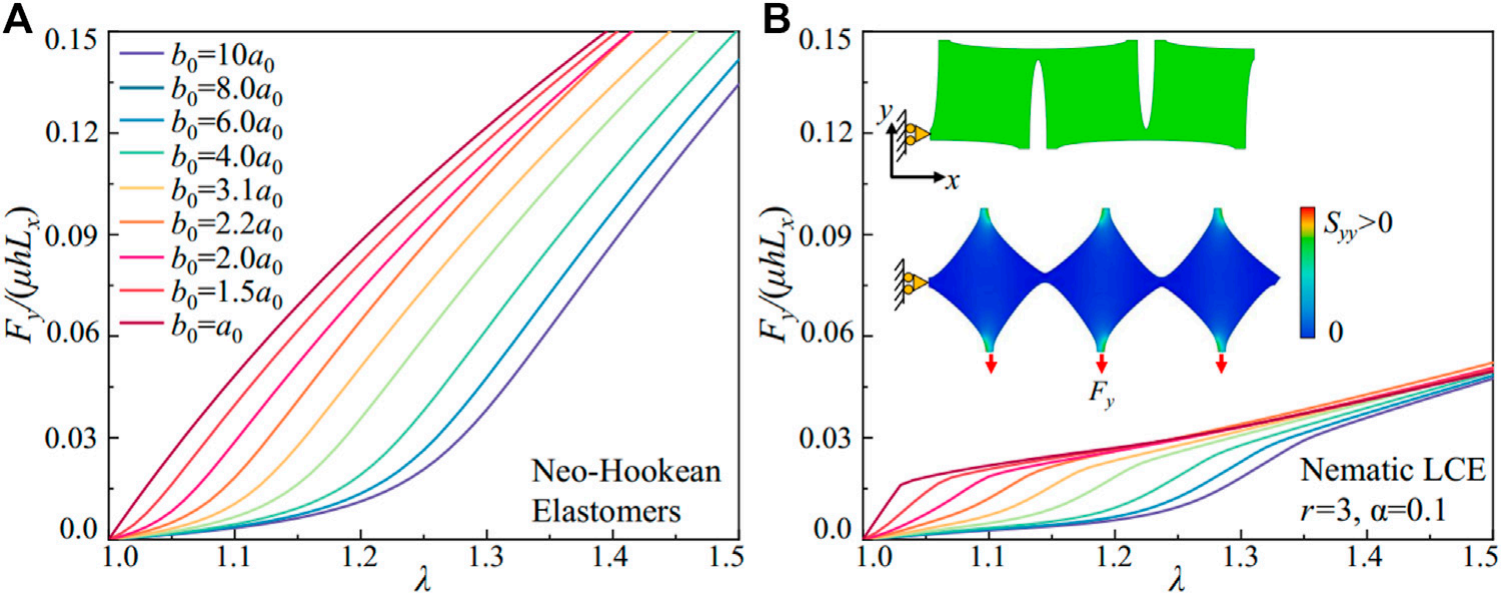
FE simulations of the metamaterials with different geometries. **(A)** The scaled stretching force *F*_*y*_/*µL*_*x*_ *h* in metamaterials composed of Neo-Hookean elastomers with pore aspect ratios *a*_*r*_ ranging from 0.1 to 1. **(B)** The scaled stretching force *F*_*y*_/*µL*_*x*_ *h* in metamaterials composed of nematic LCEs (*r* = 3 and *α* = 0.1) with pore aspect ratio *a*_*r*_ ranging from 0.1 to 1. Inset: FE models for the metamaterials at initial and deformed states.

In the FE simulations, we study the configurations shown in Figure 1A by varying aspect ratio of the pores (*a*_*r*_ = *a*_0_/*b*_0_), with a fixed width of the ligaments, *w*_0_ = 0.1*L*_0_, where *L*_0_ = *a*_0_+*b*_0_+*w*_0_ is the length of the building blocks. We have explored the mechanical responses of metamaterials composed of elastomers with different geometries, including the effect of the ligament width in previous studies (Liang and Crosby, 2020a; Liang and Crosby, 2020b). Here, we focus on the effect of the pore aspect ratio and its potential coupling with the soft elasticity in nematic LCEs. 2D plane-strain simulations (Abaqus element type CPE6H) are carried out. The simulations are built upon a single layer of three unit cells, which is selected to mirror the mechanical response of the overall structure (inset, Figure 4B). Symmetric boundary conditions about the *y* axis are applied to the left boundary, while the right is stress-free. In the metamaterials under uniaxial extension, the building blocks along the *x*-axis remain horizontal, with the right edge free from constraints. To mimic the uniaxial loading conditions in experiments, the bottom boundary is prescribed with a displacement, and the top boundary is constrained to be horizontal. As a result, the horizontal building blocks in the metamaterials remain horizontal, as shown with the deformed shapes in the inset of Figure 4B. In addition, the effect of the number of horizontal building blocks in the mechanical responses is considered minor compared to the uniaxial extension along the *y*-axis.

We measure the scaled stretching force from simulations, *F*_*y*_/*µL*_*x*_ *h*, where *L*_*x*_ is the horizontal length and *h* is the thickness of the metamaterials under uniaxial extension. We compare the stress-strain relation for metamaterials composed of Neo-Hookean elastomers and the nematic LCE with soft elasticity in Figure 4. The metamaterials with elastomers show a transition from strain-stiffening to a weakly strain-softening as the pore aspect ratio *a*_*r*_ increases from 0.1 to 1, as shown Figure 4A. For metamaterials with circular pores (*a*_*r*_ *∼*1), the internal deformation is governed by the ligament stretching, leading to a stress-strain relation similar to the constituent elastomers with a negligible softening. For metamaterials with elliptical pores (*a*_*r*_ *∼*0), the ligament undergoes a bending deformation first, inducing a compliant response with the effective shear modulus around 0.1*µ*. The subsequent ligament stretching stiffens the stress-strain curve, with a fivefold increase of the effective shear modulus to 0.5*µ*. The geometry of the pores in the building blocks of metamaterials is shown to program the mechanical responses. However, the design space is often limited, for example, by the finite size of the ligament and the maximum rotation angle in bending.

The metamaterials can bypass the limitations in geometries by incorporating the nematic LCEs with soft elasticity. In Figure 4B, we simulate the metamaterials composed of nematic LCEs (*r* = 3 and *α* = 0.1). The nematic LCE deforms with near-constant stresses as the stretch increases from 1.05 to 1.5 (Figure 2A). Integrating the building blocks with different pore shapes, the stiffness of the metamaterials with the same geometry decreases due to the rotation of the liquid crystal mesogens. However, the tunability for the mechanical properties in metamaterials is substantially increased, with an enlarged space in programming the strain-softening and strain-stiffening responses. For metamaterials with circular pores (*a*_*r*_ *∼*1), as the nematic LCE in the ligament is stretched to the critical value for soft elasticity, a dramatic softening with a tenfold reduction in the effective shear modulus (0.5*µ* to 0.05*µ*) is observed. For metamaterials with elliptical pores (*a*_*r*_ *=* 0.1), the stretch in the bending ligament can trigger the soft elasticity in the nematic LCE. Therefore, the metamaterial shows a compliant response with an effective shear modulus as low as 0.03*µ*, up to the stretch as large as 1.2. The transition from bending to stretching in the ligament leads to higher structural stiffness and larger local stretch, driving the nematic LCE to exit the soft elasticity with the stretch larger than ${\bar{\lambda }}_{2}$ (Figure 2). Therefore, the metamaterials generate a strong strain-stiffening effect with a sixfold increase in the effective shear modulus (0.03*µ* to 0.18*µ*).

**FIGURE 5:**
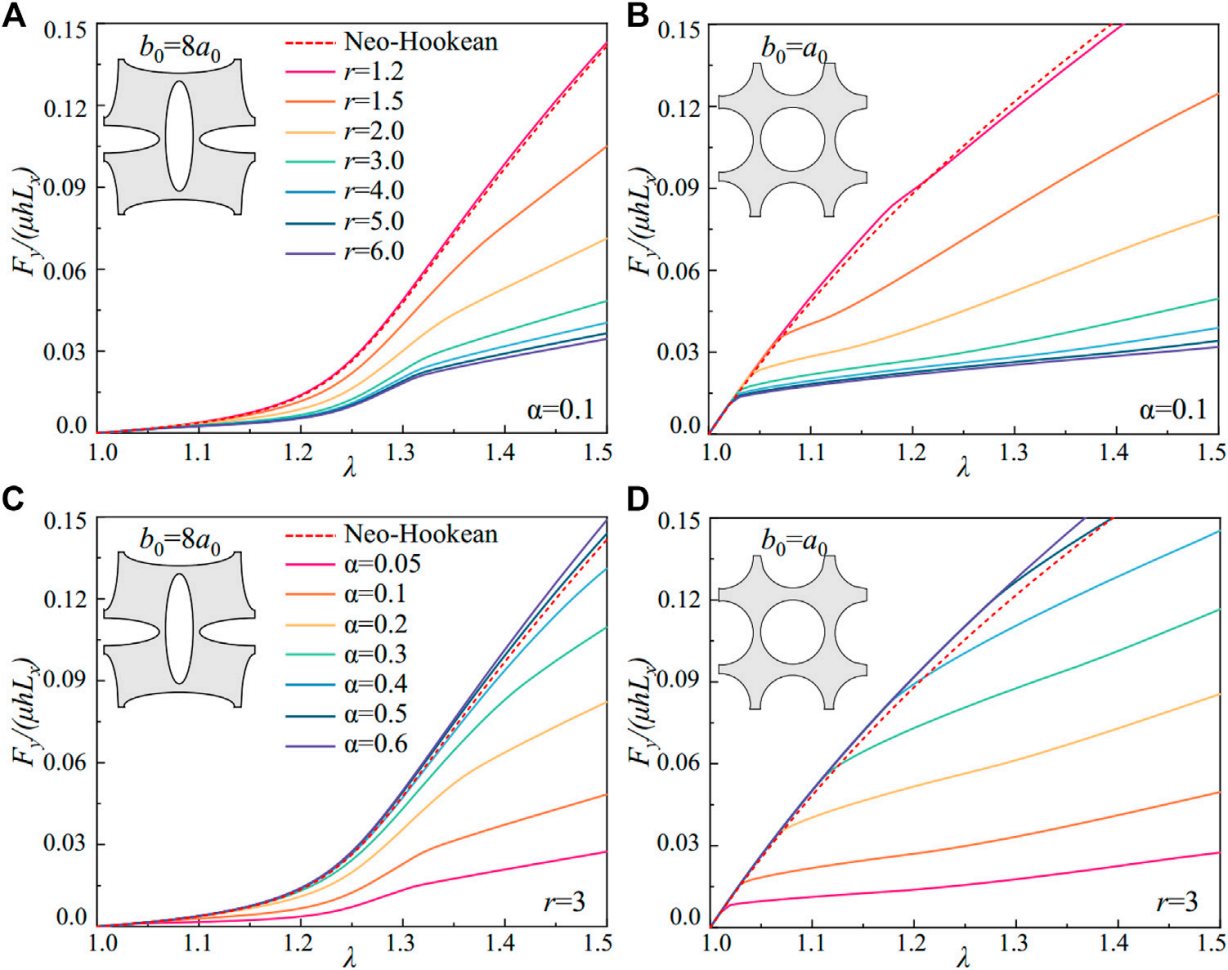
The scaled stretching force *F*_*y*_/*µL*_*x*_ *h* in LCE metamaterials under uniaxial extension. The nematic LCE with **(A)** a fixed fluctuation *α* = 0.1 and **(C)** a fixed liquid crystal ordering *r* = 3 in the metamaterials with elliptical pores (*b*_0_ = 8*a*_0_). The nematic LCE with **(B)** a fixed fluctuation *α* = 0.1 and **(D)** a fixed liquid crystal ordering *r* = 3 in the metamaterials with circular pores (*b*_0_ = *a*_0_).

We can design the stress-strain relations in metamaterials via the soft elasticity of the nematic LCE. With a fixed liquid crystal ordering fluctuation of *α* = 0.1, the anisotropy in LCE can increase from *r* = 1, requiring a larger local stretch to rotate the liquid crystal mesogens. As a result, the constitutive responses in the nematic LCE depart from the Neo-Hookean elastomer, showing the soft elasticity that lasts for a wider region in the stretch and generates lower stress in deformation (Figure 2A). As shown in Figures 5A,B, the molecular liquid crystal ordering in the LCE can program the stress-strain relation in metamaterials with different pore shapes (elliptical in Figure 5A and circular in Figure 5B). A large range of stress-strain relations is created in the metamaterials with different liquid crystal orderings (*r*). In addition, all the stress-strain curves in the LCE metamaterials fall beneath the one generated by the metamaterial composed of the Neo-Hookean elastomers (dashed line), given the reduction in the stress due to the liquid crystal mesogens’ rotation. The stress-strain curves in the LCE metamaterials start to depart from the elastomeric metamaterials after reaching the critical stretch for the onset of soft elasticity $\left({\bar{\lambda }}_{1}\right)$ with a softer response. The effective shear modulus of the metamaterials decreases with anisotropy *r*.

The stress-strain relation in metamaterials also can be designed via the fluctuations of the liquid crystal ordering in the nematic LCE. With a fixed anisotropy of the nematic LCE at *r* = 3, the spread of the liquid crystal ordering distribution increases the energy barrier for the molecular rotation of liquid crystals, suppressing the soft elasticity. Similar to controlling the constitutive responses of the nematic LCE via the liquid crystal ordering with *r*, the metamaterials composed of the nematic LCE can generate highly tunable stress-strain relations for both elliptical (Figure 5C) and circular pores (Figure 5D). Furthermore, with the increase of *α*, the stress-strain relations can recover the one generated by the metamaterial with the Neo-Hookean elastomers (dashed line). Different from varying the anisotropy in the nematic LCE with *r*, the critical stretch for the onset of soft elasticity $\left({\bar{\lambda }}_{1}\right)$ is very sensitive to the fluctuation in the liquid crystal ordering *α* (see Figures 2B,D). Therefore, the stress-strain curves for the LCE metamaterials depart from the metamaterials with Neo-Hookean elastomers at different stretches with increasing *α* (Figure 5D).

The FE simulations demonstrated that the soft elasticity in the nematic LCE could couple with the structural design in the building blocks of metamaterials, programming the stress-strain relations in uniaxial extension. As a result, the molecular control of the constitutive responses of the materials and the structural design of the geometry in the building blocks are no longer independent components in the LCE metamaterials. Instead, the two components synergetically program the mechanical properties, leading to tunable strain-softening and strain-stiffening behaviors at the required deformation and the active control of the stress-strain relations without altering the constituent materials.

## 4 Analytical Models for LCE Metamaterials

We develop an analytical model to understand how the metamaterial geometry and the nematic LCE soft elasticity affect the stress-strain relation under uniaxial extension. Since the soft elasticity in LCE is related to the local stretch in the metamaterials, we first propose a simplified kinematics analysis to describe the local deformation in the ligament.

**FIGURE 6:**
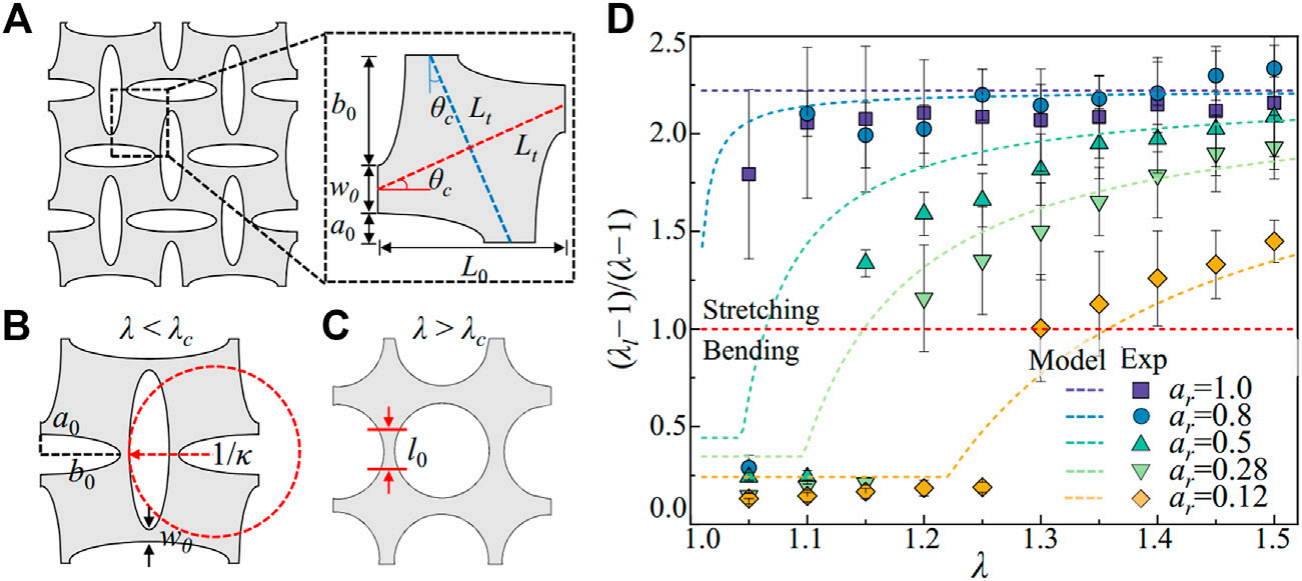
Analytical model for the local stretch in the metamaterial. **(A)** Deformation kinematics of the metamaterials under uniaxial extension. The ligament bending is reflected by the rotations in blue and red dashed lines, with a maximum bending angle *θ*_*c*_. **(B)** Measurement of the local stretch in ligament bending through the changes of the radius of curvature *κ*. **(C)** Measurement of the local stretch in the ligament stretching through the length of the deformed ligament *l*_0_. **(D)** The ratio between the local and the global stretch ( ${\bar{\lambda }}_{l}$ −1)/(*λ*−1) against the stretch in the metamaterials *λ*. The solid symbols are adopted from experiments (Liang and Crosby, 2020a). The red-dashed line separates the ligament bending and stretch deformation.

As shown in Figure 6A, the maximum angle in bending is *θ*_*c*_ = arctan ((*b*_0_−*a*_0_)/*L*_0_), where the “plates” in the building blocks are rotated along the *y*-axis (Liang and Crosby, 2020a; Liang and Crosby, 2020b). The rotation angle *θ*_*c*_ is reached with a critical stretch *λ*_*c*_ = *L*_t_/*L*_0_, where $L_{t}=\sqrt{L_{0}^{2}+{\left(b_{0}-a_{0}\right)}^{2}}$ is the distance between the center of the ligaments (marked by the dashed lines in Figure 6A). Here, we designate the global stretch applied to the metamaterial as *λ* without a bar. The maximum local strain in bending is defined as *w*_*0*_Δ*κθ*_*c*_/2, where Δ*κ* is the change of curvatures at the neck of the ligaments, $\Delta \kappa =2/\left(a_{0}+b_{0}\right)-a_{0}/b_{0}^{2}$ (Figure 6B). As *λ*<*λ*_*c*_, the rotational angle *θ* and the local stretch ${\bar{\lambda }}_{l}$ increase with the global stretch *λ* before reaching a maximum angle *θ*_*c*_. Therefore, the local stretch in ligament bending is,

$$\begin{array}{c} {\bar{\lambda }}_{l}\left(\lambda \right)=1+\frac{w_{0}\theta_{c}\left(\lambda -1\right)}{\left(\lambda_{c}-1\right)}\left(\frac{1}{a_{0}+b_{0}}-\frac{a_{0}}{2b_{0}^{2}}\right),\;\lambda <\lambda_{c}. \end{array}$$ (10)

For *λ* >*λ*_*c*_, the maximum rotational angle is *θ*_*c*_ reached, and further stretching the metamaterials leads to elongation of the ligament with a length *l*_0_ = 0.5 (*a*_0_+*b*_0_), as shown in Figure 6C. For the building blocks with a length *L*_*0*_ deform with the stretch *λ*, the local stretch in the ligament is expressed as,

$$\begin{array}{c} {\bar{\lambda }}_{l}\left(\lambda \right)=1+w_{0}\theta_{c}\left(\frac{1}{a_{0}+b_{0}}-\frac{a_{0}}{2b_{0}^{2}}\right)+\frac{2L_{0}\left(\lambda -\lambda_{c}\right)}{a_{0}+b_{0}},\lambda >\lambda_{c}. \end{array}$$ (11)

We compare the local stretch predicted by Eqs 10, 11 with previous experiments (Liang and Crosby, 2020a) in Figure 6D. The ratio between the local strain $\bar{\lambda }$ _*l*_−1 with the global strain *λ*−1 reveals how the geometry of the metamaterials controls the local deformation. For ( ${\bar{\lambda }}_{l}$ −1)/(*λ*−1)<1, the ligament undergoes the bending deformation, and the local stretch in the nematic LCE is attenuated. For ( ${\bar{\lambda }}_{l}$ −1)/(*λ*−1)>1, the ligament undergoes the stretching deformation, and the local stretch in the nematic LCE is strengthened. As shown in Figure 6D, our model captures the local strain in metamaterials with different pore shapes, showing good agreements with experiments. The ligaments in metamaterials with circular pores (*a*_*r*_ = 1 and 0.8) deform with a large local stretch under uniaxial extension, while the ligaments in metamaterials with elliptical pores (*a*_*r*_ = 0.5, 0.28, and 0.12) can bend first, followed by stretching, leading to a transition from low to high local stretch. Modulating the local stretch in the ligaments can trigger the onset and exit of the nematic LCE’s soft elasticity, enabling the mechanical properties’ synergetic programming with both material and structural responses.

**FIGURE 7:**
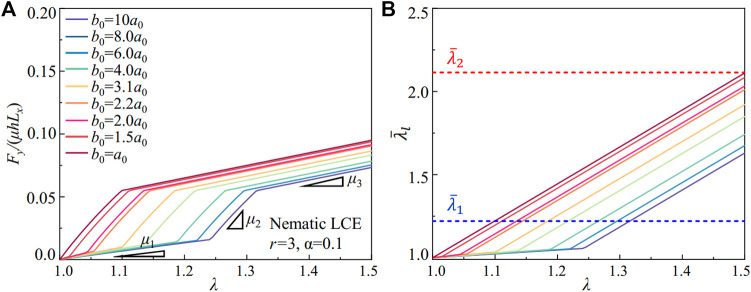
**(A)** The scaled stretching force *F*_*y*_/*µL*_*x*_ *h* predicted by the model for metamaterials with nematic LCEs (*r* = 3 and *α* = 0.1). **(B)** The local stretch in the ligament ${\bar{\lambda }}_{l}$ as the metamaterials under uniaxial extension *λ*. The critical stretches for the onset $\left({\bar{\lambda }}_{1}\right)$ and exiting $\left({\bar{\lambda }}_{2}\right)$ of the semi-soft elasticity in the materials are plotted with dashed lines.

To relate the local deformation to the force applied to the metamaterials, we substitute the local stretch predicted by Eqs 10, 11 in the constitutive model of the nematic LCE with soft elasticity in Eq. 9. Given the crosssection area of the ligament is proportional to its width *w*_0_, the scaled stretching force in the metamaterials is *F*_*y*_/*μL*_0_ *h* = *S*_*yy*_ *w*_0_/*μL*_0_. In Figure 7A, we plot the stress-strain relation predicted by the analytical model of the nematic LCE with *r* = 3 and *α* = 0.1, with the same pore shapes adopted in the FE simulations in Figure 2B. Albeit simple, our analytical model qualitatively captures the mechanical responses in the metamaterial, especially the coupling between local stretch in the ligament and the soft elasticity in the nematic LCE. The predicted stresses are larger than the FE simulations, as the inhomogeneous stress distribution in the ligament’s crosssection is neglected in the model.

The strain-softening response for the metamaterials with circular pores is predicted by considering the soft elasticity in the nematic LCE. As the pores become elliptical, the bending deformation in the ligament induces a local stretch smaller than the one for the onset of soft elasticity $\left({\bar{\lambda }}_{1}\right)$ in the nematic LCE (Figure 7B), leading to the elastic responses in the nematic LCE without liquid crystal rotations. The nematic LCE behaves as the Neo-Hookean elastomers for the local stretch ${\bar{\lambda }}_{l}$ < ${\bar{\lambda }}_{1}$ . The compliant responses (represented by *μ*_1_ in Figure 7A) in the metamaterials are caused by the bending deformation in the ligament. As the ligament stretches after rotation, the local stretch quickly increases with the uniaxial extension applied to the metamaterials, leading to stiffening responses (represented by *μ*_2_ in Figure 7A). As the local stretch is larger than ${\bar{\lambda }}_{1}$ , the nematic LCE in the ligament is controlled by the semi-soft elasticity before reaching the stretch ${\bar{\lambda }}_{2}$ (represented by *μ*_3_ in Figure 7A).

The relation between the local stretch and the global stretch determines the strain-softening and strain-stiffening responses in the metamaterials. As the global stretch *λ*_*c*_ is applied to the metamaterials, the maximum local stretch for rotation is ${\bar{\lambda }}_{l}^{c}=1+w_{0}\theta_{c}\left(\frac{1}{a_{0}+b_{0}}-\frac{a_{0}}{2b_{0}^{2}}\right)$ . The effective shear modulus is governed by ligament bending, with *μ*_1_ = *w*_0_ *S*_*yy*_ $\left({\bar{\lambda }}_{l}^{c}\right)$ /*L*_0_ (*λ*_*c*_−1), namely,

$$\begin{array}{c} \mu_{1}=\mu \frac{w_{0}}{L_{0}\left(\lambda_{c}-1\right)}\left(\lambda_{l}^{c}-{\left(\frac{1}{\lambda_{l}^{c}}\right)}^{2}\right). \end{array}$$ (12)

**FIGURE 8:**
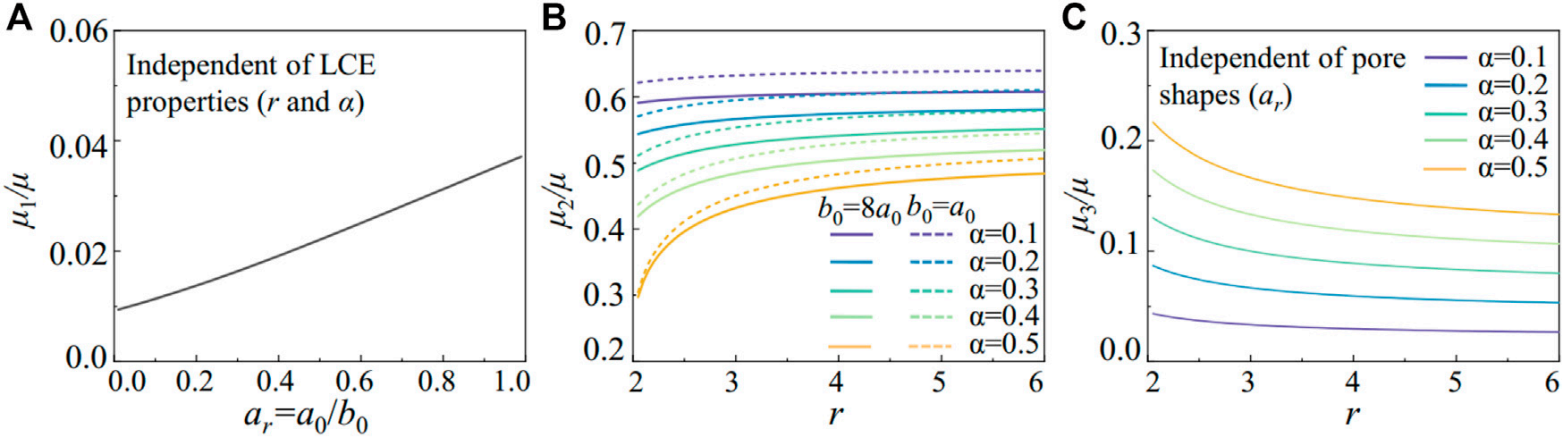
The effective shear moduli in the LCE metamaterials. **(A)** For the local stretch ${\bar{\lambda }}_{l}$ < ${\bar{\lambda }}_{l}^{c}$ , the effective shear modulus *μ*_1_ is governed by ligament bending, independent of the nematic LCE material properties (*r* and *α*). **(B)** For the local stretch ${\bar{\lambda }}_{l}^{c}$ < ${\bar{\lambda }}_{l}$ < ${\bar{\lambda }}_{1}$ , the effective shear modulus *μ*_2_ is controlled by the pore geometries and the nematic LCE material properties. **(C)** For the local stretch ${\bar{\lambda }}_{1}$ < ${\bar{\lambda }}_{l}$ , the effective shear modulus *μ*_3_ is only controlled by the nematic LCE material properties, independent of the pore shapes (*a*_*r*_).

Here, we consider the semi-soft elasticity with onset stretch ${\bar{\lambda }}_{1}$ > ${\bar{\lambda }}_{l}^{c}$ and describe the nematic LCE in the ligament with the first expression in Eq. 9. The pore shapes govern the effective shear modulus *μ*_1_ in the compliant region of the metamaterials, regardless of the nematic LCE’s properties. As shown in Figure 8A, the effective shear modulus with a fixed ligament width (*w*_0_ = 0.1*L*_0_) increases as the pores change from ellipses to circles, with *a*_*r*_ increasing from 0 to 1. A larger force is required to deform the metamaterials with circular pores as the global stretch *λ*_*c*_ to reach the maximum rotation angle reduces to 1.

The strain-stiffening responses happen as the ligament starts to stretch, where the local stretch in the nematic LCE changes from ${\bar{\lambda }}_{l}^{c}$ to ${\bar{\lambda }}_{1}$ as the metamaterials stretch from *λ*_*c*_. By setting ${\bar{\lambda }}_{l}\left(\lambda_{1}\right)={\bar{\lambda }}_{1}$ in Eq. 11, the global stretch *λ*_1_ in the metamaterials that induces the local stretch $\bar{\lambda }$ _1_ in the nematic LCE is,

$$\begin{array}{c} \lambda_{1}=\lambda_{c}+\frac{a_{0}+b_{0}}{2L_{0}}\left({\bar{\lambda }}_{1}-1-w_{0}\theta_{c}\left(\frac{1}{a_{0}+b_{0}}-\frac{a_{0}}{2b_{0}^{2}}\right)\right). \end{array}$$ (13)

The effective shear modulus follows *μ*_2_ = *w*_0_(*S*_*yy*_ $\left({\bar{\lambda }}_{1}\right)$ −(*S*_*yy*_ $\left({\bar{\lambda }}_{l}^{c}\right)$ /*L*_0_ (*λ*_1_−*λ*_*c*_), namely,

$$\begin{array}{c} \mu_{2}=\mu \frac{w_{0}}{L_{0}\left(\lambda_{1}-\lambda_{c}\right)}\left({\bar{\lambda }}_{1}-\lambda_{l}^{c}+{\left(\frac{1}{\lambda_{l}^{c}}\right)}^{2}-{\left(\frac{1}{{\bar{\lambda }}_{1}}\right)}^{2}\right). \end{array}$$ (14)

In Figure 8B, we plot the effect of soft elasticity in the nematic LCE on effective shear modulus *μ*_2_ with a fixed ligament width *w*_0_ = 0.1*L*_0_. In the metamaterials with elliptical pores (*b*_0_ = 8*a*_0_), the aligned liquid crystals (increasing anisotropy *r*) in the nematic LCE lead to a larger *μ*_2_, resulting in a stronger stiffening effect in the metamaterials. The fluctuation of the liquid crystal ordering (increasing *α*) reduces *μ*_2_, as it increases the critical stretch ${\bar{\lambda }}_{1}\;$ for the onset of soft elasticity, leading to weaker stiffening behavior. The geometry of the pores in the metamaterial also programs the stiffening response. For example, the metamaterials with the circular pore (*b*_0_ = *a*_0_) can generate larger effective shear moduli than the elliptical pores (dashed lines in Figure 8B) with higher structural stiffness.

As the nematic LCE reaches the local stretch for soft elasticity, the metamaterials deform until the ligament reaches the critical local stretch ${\bar{\lambda }}_{2}$ , where the global stretch *λ*_2_ is applied. By adopting the constitutive relation for the semi-soft elasticity in Eq. 9, the effective shear modulus defined as *μ*_3_ = *w*_0_(*S*_*yy*_ $\left({\bar{\lambda }}_{2}\right)$ −(*S*_*yy*_ $\left({\bar{\lambda }}_{1}\right)$ /*L*_0_ (*λ*_2_−*λ*_1_) can be expressed,

$$\begin{array}{c} \mu_{3}=\mu \frac{2w_{0}}{a_{0}+b_{0}}\left(1-\frac{1}{{\bar{\lambda }}_{1}^{3}}\right). \end{array}$$ (15)

As shown in Eq. 15, the effective shear modulus is independent of the pore shapes, where 2*w*_0_/(*a*_0_+*b*_0_) = 2*w*_0_/(*L*_0_-*w*_0_). The effective shear modulus *μ*_3_ is only controlled by the ligament width and critical stretch ${\bar{\lambda }}_{1}\;$ for the onset of soft elasticity. In Figure 8C, we plot the effective shear moduli *μ*_3_ by varying the nematic LCE properties with *r* and *α*, with a fixed ligament width (*w*_0_ = 0.1*L*_0_). The metamaterials with different pore shapes follow the same master curve for *μ*_3_ (also found in the FE simulations in Figure 4B for stretch larger than 1.4), which increases with ${\bar{\lambda }}_{1}$ as indicated by Eq. 15. Therefore, reducing the liquid crystal ordering with smaller *r* (Figure 2B) or increasing the fluctuation of the ordering with larger *α* (Figure 2D) leads to a larger *μ*_3_. With the analytical developed above, we can design the strain-softening and stiffening responses by controlling the liquid crystal ordering at the molecular level and the building blocks’ geometry at the structural level.

**FIGURE 9:**
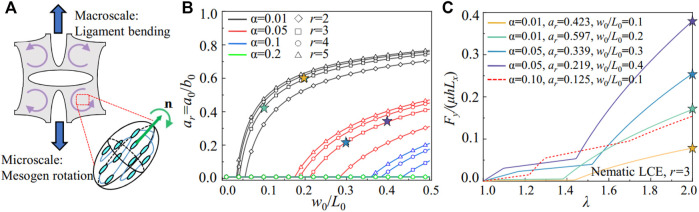
**(A)** Schematic of macroscale ligament bending and the microscale mesogen rotation. **(B)** The geometries of the metamaterials required for the continuous transition from the ligament bending to the soft elasticity in the nematic LCE, with different material properties. **(C)** The scaled stretching force *F*_*y*_/*µL*_*x*_ *h* in LCE metamaterials with the continuous transition of ${\bar{\lambda }}_{l}^{c}$ = ${\bar{\lambda }}_{1}$ . The red dashed line represents the LCE metamaterial without the continuous transition.

Finally, we can join the deformation induced by the ligament bending and the LCE soft elasticity with ${\bar{\lambda }}_{l}^{c}$ = ${\bar{\lambda }}_{1}$ to design the compliant responses in LCE metamaterials. As demonstrated in Figure 9A, by designing the geometry of the building blocks and the molecular structures of the LCE, the macroscale ligament bending and the microscale mesogen rotation can be tuned synergetically. The nematic LCE can change continuously from a low-stretch (soft) bending deformation to the soft elasticity in the materials without causing the stiffening effect in *μ*_2_. The critical conditions can be reached by controlling the molecular compositions in the LCE and the geometry of the building blocks via,

$$\begin{array}{c} \left[{\left(1+w_{0}\theta_{c}\left(\frac{1}{a_{0}+b_{0}}-\frac{a_{0}}{2b_{0}^{2}}\right)\right)}^{3}-1\right]\left(r-1\right)-\alpha {\left(1+w_{0}\theta_{c}\left(\frac{1}{a_{0}+b_{0}}-\frac{a_{0}}{2b_{0}^{2}}\right)\right)}^{3}r=0. \end{array}$$ (16)

In Figure 9B, we plot the critical geometric conditions (*a*_*r*_ and *w*_*0*_/*L*_*0*_) based on Eq. 16 for different nematic LCE properties (*r* and *α*). Since the ligament bending deformation tends to attenuate the local stretch, a low fluctuation (small *α*) or a high anisotropy (large *r*) can reduce the critical stretch for the onset of soft elasticity that enables the continuous transition of the compliant responses between the ligament bending and soft elasticity. We compare the stress-strain curves for the LCE metamaterials with the anisotropy *r* = 3 with the continuous transition (marked by stars in Figure 9B) to the discontinuous one adopted from Figure 7A. As shown in Figure 9C, the LCE metamaterials with the continuous transition generate the compliant responses up to a stretch of 1.4 (solid lines), much larger than the discontinuous one (1.2 in the dashed line). In addition, the effective shear modulus (slope of the compliant region) is greatly reduced for the similar ligament width due to the soft elasticity in the nematic LCE. Particularly, for the nematic LCE with *r* = 3 and *α =* 0.01, the metamaterials with the geometric conditions *a*_*r*_ = 0.423 and *w*_*0*_/*L*_*0*_ = 0.1 can generate an elastic response with near-zero stiffness. The subsequent stiffening responses in the metamaterials also strengthen as the nematic LCE exits the soft elasticity and generates large stress in the ligament with the local stretch concentration. The analytical model proposed here connects the deformation of the building blocks and the soft elasticity of the nematic LCE via the local stretch, guiding the program of the stress-strain relations in the metamaterials.

## 5 Conclusion

In conclusion, we demonstrate the material-based programmability enabled by metamaterials composed of nematic LCEs, especially the stress-strain relations with tunable strain-softening and strain-stiffening effects. Taking advantage of the tunable bending and stretching deformation enabled by the geometry of the building blocks and the semi-soft elasticity of the nematic LCE, we engineer the local stretch in the ligament of the metamaterials to program the stress-strain relation in the metamaterial under uniaxial extension. Starting from the molecular description of the soft elasticity, we relate the liquid crystal ordering to the constitutive model of the nematic LCE and integrate the semi-soft elasticity of LCE into the metamaterials. Numerical simulations have revealed that the attenuated local stretch due to the ligament bending and the soft elasticity in the nematic LCE induce a compliant response in the metamaterials, where the effective shear modulus is much lower than the constituent materials. The subsequent stretching deformation in the ligament and the exiting of the soft elasticity in the nematic LCE leads to the surge of the stress, leading to a stiffening response. To relate the softening and stiffening responses to the geometric and material parameters in the LCE metamaterials, we develop an analytical model that predicts the local stretch in the ligament and calculates the force in the LCE metamaterials. The metamaterials undergo a transition between strain-softening and strain-stiffening with different effective shear moduli, depending on the maximum local stretch in bending and the critical stretch for the onset of the soft elasticity. By designing a continuous transition from the ligament bending to the semi-soft elasticity in the LCE, we can program an elastic region with near-zero stiffness up to the stretch of 1.4. The highly programmable softening and stiffening behaviors offer a material-based control of the mechanical properties in the LCE metamaterials, inducing the stress-strain relations within the space prescribed by the Neo-Hookean elastomers with the same shear modulus. The LCE metamaterials provide a platform for material-based programmability, facilitating the development of advanced forms of mechanical metamaterials and impacting the design of robotic systems.

## Data Availability

The original contributions presented in the study are included in the article/Supplementary Material, further inquiries can be directed to the corresponding author.

## References

[B1] Bai R. Bhattacharya K. (2020). Photomechanical Coupling in Photoactive Nematic Elastomers. J. Mech. Phys. Sol. 144, 104115. 10.1016/j.jmps.2020.104115

[B2] Bertoldi K. Vitelli V. Christensen J. Van Hecke M. (2017). Flexible Mechanical Metamaterials. Nat. Rev. Mater. 2, 1–11. 10.1038/natrevmats.2017.66

[B3] Biggins J. S. Terentjev E. M. Warner M. (2008). Semisoft Elastic Response of Nematic Elastomers to Complex Deformations. Phys. Rev. E Stat. Nonlin Soft Matter Phys. 78, 041704. 10.1103/PhysRevE.78.041704 18999442

[B4] Biggins J. S. Warner M. Bhattacharya K. (2012). Elasticity of Polydomain Liquid crystal Elastomers. J. Mech. Phys. Sol. 60, 573–590. 10.1016/j.jmps.2012.01.008

[B5] Bladon P. Terentjev E. M. Warner M. (1993). Transitions and Instabilities in Liquid crystal Elastomers. Phys. Rev. E 47, R3838–R3840. 10.1103/physreve.47.r3838 9960562

[B6] Chen T. Pauly M. Reis P. M. (2021). A Reprogrammable Mechanical Metamaterial with Stable Memory. Nature 589, 386–390. 10.1038/s41586-020-03123-5 33473228

[B7] Coulais C. Overvelde J. T. Lubbers L. A. Bertoldi K. Van Hecke M. (2015). Discontinuous Buckling of Wide Beams and Metabeams. Phys. Rev. Lett. 115, 044301. 10.1103/PhysRevLett.115.044301 26252687

[B8] Dykstra D. M. Busink J. Ennis B. Coulais C. (2019). Viscoelastic Snapping Metamaterials. J. Appl. Mech. 86, 1. 10.1115/1.4044036

[B9] Fleck N. A. Deshpande V. S. Ashby M. F. (2010). Micro-architectured Materials: Past, Present and Future. Proc. R. Soc. A. 466, 2495–2516. 10.1098/rspa.2010.0215

[B10] Gardel M. L. Shin J. H. Mackintosh F. C. Mahadevan L. Matsudaira P. Weitz D. A. (2004). Elastic Behavior of Cross-Linked and Bundled Actin Networks. Science 304, 1301–1305. 10.1126/science.1095087 15166374

[B11] Herbert K. M. Fowler H. E. Mccracken J. M. Schlafmann K. R. Koch J. A. White T. J. (2021). Synthesis and Alignment of Liquid Crystalline Elastomers. Nat. Rev. Mater. 7, 1–16. 10.1038/s41578-021-00359-z

[B12] Kadic M. Milton G. W. Van Hecke M. Wegener M. (2019). 3D Metamaterials. Nat. Rev. Phys. 1, 198–210. 10.1038/s42254-018-0018-y

[B13] Kim J. Zhang G. Shi M. Suo Z. (2021). Fracture, Fatigue, and Friction of Polymers in Which Entanglements Greatly Outnumber Cross-Links. Science 374, 212–216. 10.1126/science.abg6320 34618571

[B14] Kochmann D. M. Bertoldi K. (2017). Exploiting Microstructural Instabilities in Solids and Structures: from Metamaterials to Structural Transitions. Appl. Mech. Rev. 69. 10.1115/1.4037966

[B15] Küupfer J. Finkelmann H. (1994). Liquid crystal Elastomers: Influence of the Orientational Distribution of the Crosslinks on the Phase Behaviour and Reorientation Processes. Macromolecular Chem. Phys. 195, 1353–1367. 10.1002/macp.1994.021950419

[B16] Lake G. Thomas A. (1967). The Strength of Highly Elastic Materials. Proc. R. Soc. Lond. Ser. A. Math. Phys. Sci. 300, 108–119. 10.1098/rspa.1967.0160

[B17] Lakes R. (1987). Foam Structures with a Negative Poisson's Ratio. Science 235, 1038–1040. 10.1126/science.235.4792.1038 17782252

[B18] Launey M. E. Buehler M. J. Ritchie R. O. (2010). On the Mechanistic Origins of Toughness in Bone. Annu. Rev. Mater. Res. 40, 25–53. 10.1146/annurev-matsci-070909-104427

[B19] Liang X. Crosby A. J. (2020a). Programming Impulsive Deformation with Mechanical Metamaterials. Phys. Rev. Lett. 125, 108002. 10.1103/physrevlett.125.108002 32955335

[B20] Liang X. Crosby A. J. (2020b). Uniaxial Stretching Mechanics of Cellular Flexible Metamaterials. Extreme Mech. Lett. 35, 100637. 10.1016/j.eml.2020.100637

[B21] Liang X. Fu H. Crosby A. J. (2022). Phase-transforming Metamaterial with Magnetic Interactions. Proc. Natl. Acad. Sci. USA 119, e2118161119. 10.1073/pnas.2118161119 34983853 PMC8740733

[B22] Meyers M. A. Chawla K. K. (2008). Mechanical Behavior of Materials. United Kingdom: Cambridge University Press.

[B23] Meyers M. A. Chen P.-Y. Lin A. Y.-M. Seki Y. (2008). Biological Materials: Structure and Mechanical Properties. Prog. Mater. Sci. 53, 1–206. 10.1016/j.pmatsci.2007.05.002 19627786

[B24] Pishvar M. Harne R. L. (2020). Foundations for Soft, Smart Matter by Active Mechanical Metamaterials. Adv. Sci. 7, 2001384. 10.1002/advs.202001384 PMC7509744 32999844

[B25] Popov Y. O. Semenov A. N. (1998). Nematic Ordering in Anisotropic Elastomers: Effect of Frozen Anisotropy. Eur. Phys. J. B 6, 245–256. 10.1007/s100510050547

[B26] Quan H. Yang W. Lapeyriere M. Schaible E. Ritchie R. O. Meyers M. A. (2020). Structure and Mechanical Adaptability of a Modern Elasmoid Fish Scale from the Common Carp. Matter 3, 842–863. 10.1016/j.matt.2020.05.011

[B27] Schaedler T. A. Jacobsen A. J. Torrents A. Sorensen A. E. Lian J. Greer J. R. (2011). Ultralight Metallic Microlattices. Science 334, 962–965. 10.1126/science.1211649 22096194

[B28] Sonnet A. M. Virga E. G. (2012). Dissipative Ordered Fluids: Theories for Liquid Crystals. Berlin, Germany: Springer Science & Business Media.

[B29] Storm C. Pastore J. J. Mackintosh F. C. Lubensky T. C. Janmey P. A. (2005). Nonlinear Elasticity in Biological Gels. Nature 435, 191–194. 10.1038/nature03521 15889088

[B30] Surjadi J. U. Gao L. Du H. Li X. Xiong X. Fang N. X. (2019). Mechanical Metamaterials and Their Engineering Applications. Adv. Eng. Mater. 21, 1800864. 10.1002/adem.201800864

[B31] Verwey G. C. Warner M. (1997a). Compositional Fluctuations and Semisoftness in Nematic Elastomers. Macromolecules 30, 4189–4195. 10.1021/ma961801i

[B32] Verwey G. C. Warner M. (1997b). Nematic Elastomers Cross-Linked by Rigid Rod Linkers. Macromolecules 30, 4196–4204. 10.1021/ma961802a

[B33] Verwey G. C. Warner M. Terentjev E. M. (1996). Elastic Instability and Stripe Domains in Liquid Crystalline Elastomers. J. Phys. France 6, 1273–1290. 10.1051/jp2:1996130

[B34] Wang Y. C. Lakes R. S. (2005). Composites with Inclusions of Negative Bulk Modulus: Extreme Damping and Negative Poisson's Ratio. J. Compos. Mater. 39, 1645–1657. 10.1177/0021998305051112

[B35] Warner M. Terentjev E. M. (2007). Liquid crystal Elastomers. United Kingdom: Oxford University Press.

[B36] Yu X. Zhou J. Liang H. Jiang Z. Wu L. (2018). Mechanical Metamaterials Associated with Stiffness, Rigidity and Compressibility: A Brief Review. Prog. Mater. Sci. 94, 114–173. 10.1016/j.pmatsci.2017.12.003

[B37] Zhang Y. Xuan C. Jiang Y. Huo Y. (2019). Continuum Mechanical Modeling of Liquid crystal Elastomers as Dissipative Ordered Solids. J. Mech. Phys. Sol. 126, 285–303. 10.1016/j.jmps.2019.02.018

[B38] Zhou H. Bhattacharya K. (2021). Accelerated Computational Micromechanics and its Application to Polydomain Liquid crystal Elastomers. J. Mech. Phys. Sol. 153, 104470. 10.1016/j.jmps.2021.104470

